# Assimilation, Accumulation, and Metabolism of Dinophysistoxins (DTXs) and Pectenotoxins (PTXs) in the Several Tissues of Japanese Scallop *Patinopecten yessoensis*

**DOI:** 10.3390/toxins7124870

**Published:** 2015-12-01

**Authors:** Ryoji Matsushima, Hajime Uchida, Satoshi Nagai, Ryuichi Watanabe, Michiya Kamio, Hiroshi Nagai, Masaki Kaneniwa, Toshiyuki Suzuki

**Affiliations:** 1 National Research Institute of Fisheries Science, Fisheries Research Agency, 2-12-4 Fukuura, Kanazawa, Yokohama, Kanagawa 236-8648, Japan; matsur@affrc.go.jp (R.M.); huchida@affrc.go.jp (H.U.); snagai@affrc.go.jp (S.N.); rwatanabe@affrc.go.jp (R.W.); mskknnw@affrc.go.jp (M.K.); 2 Department of Ocean Sciences, Tokyo University of Marine Science and Technology, 4-5-7 Konan, Minato-ku, Tokyo 108-8477, Japan; mkamio@kaiyodai.ac.jp (M.K.); nagai@kaiyodai.ac.jp (H.N.)

**Keywords:** diarrhetic shellfish toxins, accumulation, pectenotoxin, dinophysistoxin, Japanese scallops, *Dinophysis fortii*, feeding experiment, LC/MS/MS

## Abstract

Japanese scallops, *Patinopecten yessoensis*, were fed with the toxic dinoflagellate *Dinophysis fortii* to elucidate the relative magnitude of assimilation, accumulation, and metabolism of diarrhetic shellfish toxins (DSTs) and pectenotoxins (PTXs). Three individual scallops were separately exposed to cultured *D*. *fortii* for four days. The average cell number of *D*. *fortii* assimilated by each individual scallop was 7.7 × 10^5^. Dinophysistoxin-1 (DTX1), pectenotoxin-2 (PTX2) and their metabolites were analyzed by liquid chromatography tandem mass spectrometry (LC/MS/MS) and the toxin content in individual tissues (digestive gland, adductor muscle, gill, gonad, mantle, and the others), feces and the seawater medium were quantified. Toxins were almost exclusively accumulated in the digestive gland with only low levels being detected in the gills, mantles, gonads, and adductor muscles. DTX1 and PTX2 were the dominant toxins in the *D. fortii* cells fed to the scallops, whereas the dominant toxins detected in the digestive gland of scallops were PTX6 and esterified acyl-*O*-DTX1 (DTX3). In other tissues PTX2 was the dominant toxin observed. The ratio of accumulated to assimilated toxins was 21%–39% and 7%–23% for PTXs and DTXs respectively. Approximately 54%–75% of PTX2 and 52%–70% of DTX1 assimilated by the scallops was directly excreted into the seawater mainly without metabolic transformation.

## 1. Introduction

Diarrhetic shellfish poisoning (DSP) is a severe gastrointestinal illness caused by the consumption of shellfish contaminated with diarrhetic shellfish toxins (DSTs) [1]. Based on their structures, DSTs were initially classified into three groups, okadaic acid (OA)/dinophysistoxin (DTX) analogues, pectenotoxins (PTXs), and yessotoxins (YTXs) [2,3]. OA and its analogues, dinophysistoxin-1 (DTX1) and -2 (DTX2), are the most important toxins due to their causing severe diarrhea. These toxins have been shown to be potent phosphatase inhibitors [4], a property that can cause inflammation of the intestinal tract and diarrhea [5], possibly leading to tumor promotion [6]. Because PTXs and YTXs do not cause diarrhea [7,8,9,10,11,12], they are now not considered to be members of the DST group. Because OA/DTXs and PTXs are produced by toxic dinoflagellate *Dinophysis* spp., OA/DTXs are regulated together with PTXs in European Union (EU). The regulatory level of sum of OA/DTXs and PTXs in EU is 0.16 mg/kg. The regulatory level of YTXs in EU is 3.75 mg/kg. On the other hand, the regulatory level of OA/DTXs suggested by CODEX is 0.16 mg/kg. With the change in the definition of DSTs in Japan in April 2015, the MBA as the Japanese official testing method for DSTs was replaced to instrumental methods including LC/MS/MS to detect OA analogues exclusively on the CODEX regulatory level. The regulation in Japan is the same as that of the US. 

**Figure 1:**
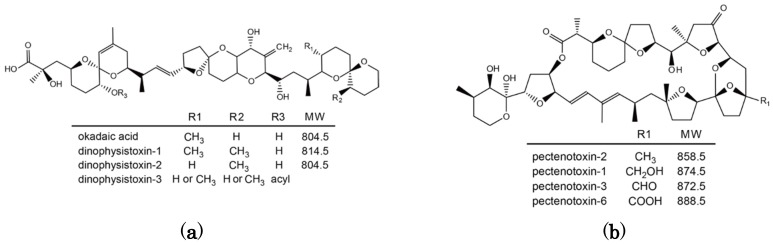
Chemical structure of okadaic acid/dinophysistoxins (**a**) and pectenotoxins (**b**).

The Japanese scallop (*Patinopecten yessoensis*) is an important aquaculture species in Japan in terms of the annual quantity of production and contamination with DSTs and other lipophilic toxins is a serious industrial problem. DST contamination of Japanese scallops is caused by feeding on toxic dinoflagellates (*Dinophysis* spp.) that produce OA, DTX1 and PTX2 [13,14,15,16] (Figure 1).

OA and DTX1 are metabolized to the esterified toxin 7-*O*-acyl-OA/DTX1 in many bivalve species including Japanese scallops [17,18]. They are collectively called dinophysistoxin-3 (DTX3). PTX2 is enzymatically hydrolyzed to PTX2 seco acid in many bivalve species [10,19,20,21], but in *P. yessoensis* PTX2 is oxidatively metabolized to pectenotoxin-1 (PTX1), pectenotoxin-3 (PTX3), and pectenotoxin-6 (PTX6) [22,23]. PTX6 is the dominant toxin that accumulates in *P. yessoensis* and this particular mode of metabolism has only been observed in this species [24].

Although quantification of toxicity of individual tissues of *P. yessoensis* by the mouse bioassay (MBA) has demonstrated that the toxins appear to be mainly accumulated in the digestive gland [1], a detailed investigation on the distribution of DSTs and other lipophilic toxins in individual tissues of Japanese scallops has not been carried out. In a previous study, the absorption efficiency of DTX1 by the digestive gland of *P. yessoensis* was estimated at less than 3% of the total amount of DTX1 fed to the scallops in *D*. *fortii* cells [17]. When a mixture of OA, DTX1, PTX6, and YTX was injected into the digestive gland, less than 20% was retained although the residual relative amount of PTX6 was slightly higher than that of OA and DTX1 [18]. Bay scallops (*Argopecten irradians*) exposed to cultured cells of *Prorocentrum lima* showed a toxin-assimilation efficiency in the scallop tissues of less than 1% [25]. Despite these investigations on the accumulation and metabolism of DSTs and other lipophilic toxins in scallops over the past twenty years, details on the accumulation kinetics of each toxin in individual tissues has not been clarified due to the inability to culture *Dinophysis* species and inadequate analytical techniques.

In this study, we describe for the first time the detailed assimilation, accumulation, and metabolism of DSTs and PTXs in individual tissues of *P. yessoensis*. For unambiguous understanding of our research, terms in our present study are defined as follows:

Assimilation: Cells/toxins that are filtered from the water.

Accumulated toxins: Toxins and their metabolites that are incorporated into tissues.

Excretion: Toxins that are simply not retained/accumulated by the organism and pass back into the water with or without metabolism.

## 2. Results

### 2.1. Feeding Experiments 

**Figure 2:**
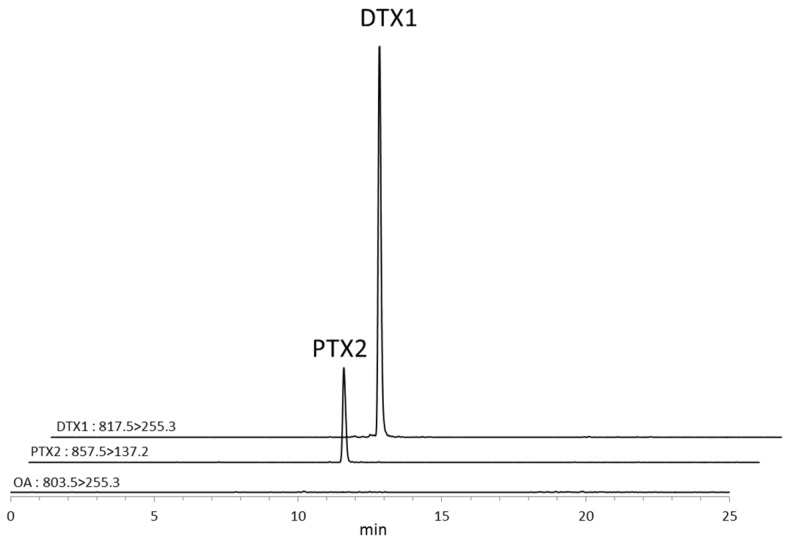
MRM LC/MS/MS chromatogram obtained from cultured *Dinophysis fortii* isolated in a coastal area in Japan.

The MRM LC/MS/MS chromatogram obtained from cultured *D. fortii* collected on the fourth day is shown in Figure 2. DTX1 and PTX2 were detected in *D. fortii*. When *D. fortii* extracts were hydrolyzed, there was no significant increase in the DTX1 content indicating that esterified DTX1 was not present in *D. fortii*.

The cell numbers and cellular toxin content of *D. fortii* fed to scallops are listed in Table 1. Although the ratio of PTX2 to DTX1 in the cellular toxins was fairly constant at between 1.9 and 2.2, the cellular toxin content of both PTX2 and DTX1 increased by the third day of cultivation. The highest cellular toxin content observed on the 3rd day was approximately eight times higher than those observed on the first day. Each individual scallop was exposed to totally 9.0 × 10^5^ cells. The total amounts of PTX2 and DTX1 exposed to each individual scallop were 55.4 and 28.2 μg, respectively.

**Table 1:** Cell numbers of *D. fortii* fed to scallops and cellular toxin contents.

Feeding Dates	Number of Cells (cells/mL)	Feeding Dose (mL)	PTX2 (pg/cell)	DTX1 (pg/cell)	Total Cell Number (cells)	Total PTX2 (μg)	Total DTX1 (μg)
1st day	1700	30 × 3	12.32	5.60	153,000	1.88	0.86
2nd day	1100	30 × 6	49.83	25.50	198,000	9.87	5.05
3rd day	1200	30 × 6	97.61	48.53	216,000	21.08	10.48
4th day	1850	30 × 6	67.67	35.37	333,000	22.53	11.78
Subtotal	-	630	-	-	900,000	55.36	28.17

The remaining cell numbers observed in seawater post-feeding of the scallops each day and the total cell numbers assimilated by each individual scallop are shown in Table 2. Seawater in which each scallop was kept contained very few *D*. *fortii* cells except for on the evening on the fourth day. The total cell numbers consumed by each individual scallop were very similar with an average of approximately 7.7 × 10^5^ *D. fortii* cells over four days. The total amounts of PTX2 and DTX1 assimilated by each individual scallop, calculated from consumed cell numbers and their cellular toxin content, were 45.3–47.5 μg and 22.9–24.0 μg, respectively.

**Table 2:** *Dinophysis* cells (cells/mL) found in seawater in post-feeding to each scallop #1, 2, 3 [remained total cell numbers].

Scallops	2nd Morning	3rd Morning	4th Morning	4th Evening	Total Cells Assimilated by Each Scallop	PTX2 (µg)	DTX1 (µg)
#1 male	0	0	13 [8,840]	200 [136,000]	755,160	45.30	22.93
#2 female	0	1 [680]	0	193 [131,240]	768,080	46.44	23.51
#3 male	0	0	0	170 [115,600]	784,400	47.54	24.08
Average	-	-	-	-	769,213	46.43	23.51

Remained total cell numbers were calculated as [(500 + 30 × 6) × remained cells].

### 2.2. Toxin Content and Profiles in Scallop Tissues

**Figure 3:**
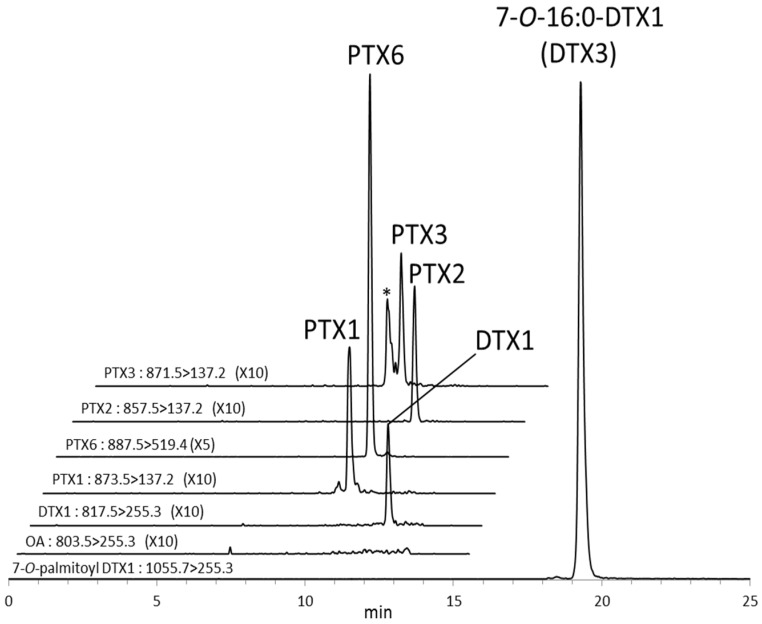
MRM LC/MS/MS chromatogram obtained from the digestive gland of the scallop #3 fed with *D. fortii*. * Hemiacetal form of PTX3.

The LC/MS/MS chromatogram obtained from the digestive gland of scallop #3 fed with *D. fortii* is shown in Figure 3. Besides DTX1 and PTX2, their metabolites 7-*O*-16:0-DTX1 (DTX3) and PTX1,3,6 were detected in the digestive gland. Because PTX3 and the hemiacetal form of PTX3 (involving addition of methanol to the aldehyde moiety) were separated, quantification of PTX3 was measured as the sum of both peak areas.

**Figure 4:**
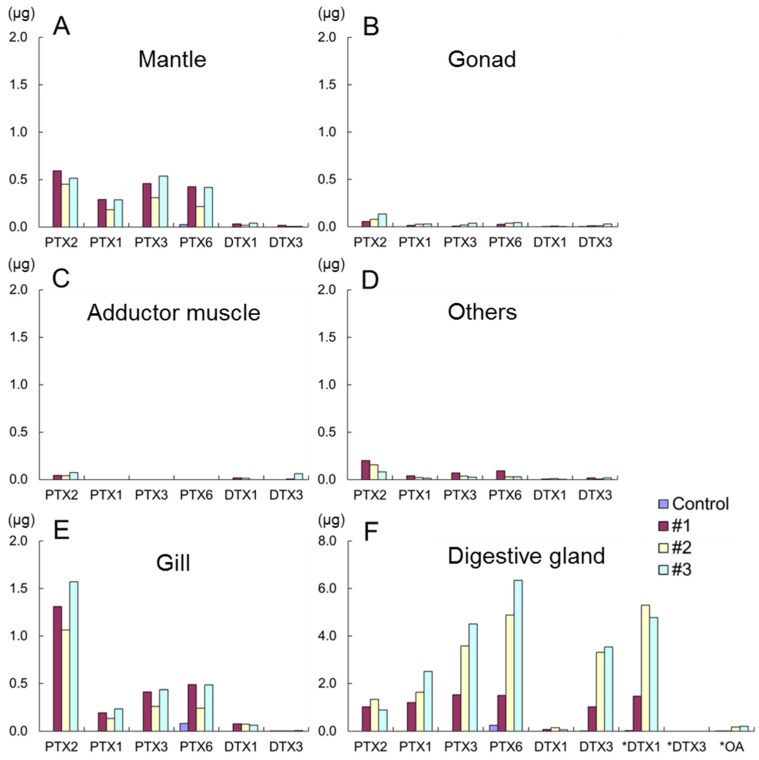
PTXs and DTXs contents in scallops #1–3 and control tissues (**A**) mantle; (**B**) gonad; (**C**) adductor muscle; (**D**) others; (**E**) gill; (**F**); digestive gland. Toxins indicated with an asterisk were obtained for post-hydrolysis extracts. DTX3; 16:0-*O*-DTX1.

The toxin content of each tissue of scallops fed with *D. fortii* cells are shown in Figure 4. The quantity of toxin in the digestive gland of the scallops was much higher than that observed in other tissues. The toxin content of tissues was in descending order: digestive gland > gill > mantle > gonad > adductor muscle. A trace level of PTX6 was observed in gill and digestive gland of control scallop.

It is noteworthy that the concentration of PTXs in the digestive gland increased with oxidization so that; PTX6 > PTX3 > PTX1 > PTX2 (Figure 4F). DTX3 concentrations in the digestive gland were also significantly higher than those of DTX1. These trends were only observed in the digestive gland. The increase of the DTX1 concentration in the digestive gland samples subjected to alkaline hydrolysis was higher than that obtained by direct quantification of DTX3 (7-*O*-16:0-DTX1) (Figure 4F). Because of the low DTX3 concentrations in other tissues, quantification of DTX1 by hydrolysis was not carried out. In contrast to the digestive gland, PTX2 was the dominant PTX analogue in other tissues.

The accumulation levels (%) of toxins in the digestive gland and other tissues, calculated from the toxin contents in each tissue and amounts of assimilated toxins, are listed in Table 3. Because we did not detect OA in the *D. fortii* strain used in our study and only low levels of OA were detected in post-hydrolysis extracts of the digestive gland, DTX concentrations are presented as the sum of DTX1 and DTX3, in the same manner as the other data. Higher concentrations of PTXs were accumulated than those of DTXs (Table 3 and Figure 4) especially in other tissues than digestive gland.

**Table 3:** Total amounts of toxins and accumulation rates in digestive gland and other tissues.

Scallops		PTX2	PTX1	PTX3	PTX6	PTXs	Accumulation Rate of PTXs (%)	DTX1	DTX3	OA	DTXs	Accumulation Rate of DTXs (%)
Digestive gland (µg)	#1	1.02	1.20	1.52	1.24	4.98	10.99	1.46 *	0.00 *	0.00 *	1.46	6.36
	#2	1.33	1.64	3.58	4.62	11.17	24.05	5.27 *	0.00 *	0.17 *	5.27	22.41
	#3	0.88	2.50	4.50	6.08	13.96	29.36	4.75 *	0.00 *	0.20 *	4.75	19.72
Other tissues (µg)	#1	2.19	0.53	0.94	0.91	4.57	10.08	0.13	0.04	0.00	0.17	0.74
	#2	1.78	0.35	0.62	0.41	3.16	6.80	0.11	0.02	0.00	0.13	0.55
	#3	2.36	0.56	1.03	0.86	4.81	10.11	0.10	0.11	0.00	0.22	0.91

* OA, DTX1 and DTX3 contents of digestive gland were obtained from post-hydrolysis extracts.

The total accumulation of PTXs and DTXs in whole scallop tissues obtained from the sum of the toxin content in the digestive gland and other tissues was 20%–40% and 7%–23%, respectively (Table 3). The total accumulation of PTXs was higher than that of DTXs. Significant difference between the total accumulation of PTXs and DTXs in other tissues was obtained by Student’s *t*-test although it was not obtained in the digestive glands. When the toxin content of the three individual scallops were compared, small differences were observed in the mantle and the gill (Figure 4A,E), but in the digestive gland of scallop #1 the toxin content was lower than those of other scallops #2 and 3 (Table 3 and Figure 4F).

### 2.3. Toxin Concentrations and Profiles in Seawater and Feces

Toxin concentrations in the seawater, in which each individual scallop were kept, are listed in Table 4. Toxins excreted directly from *D. fortii* cells to the seawater are not likely to contribute to the toxins in the seawater because toxins were hardly detected in the culture medium of *D. fortii* in our present study. Because few *D. fortii* cells were observed (except for the fourth evening; Table 2), toxins detected in the seawater are presumed to be due to excretion from scallops fed with *D. fortii.* PTX2 and DTX1 were detected in the seawater as the principal toxins.

**Table 4:** Total amount (µg) of toxins in seawaters.

Scallops	PTX2	PTX1	PTX3	PTX6	PTXs	DTX1	DTX3	OA	DTXs
#1									
2nd morning	0.91	0.09	0.12	0.02	-	2.23	0.00	0.01	-
3rd morning	8.16	0.77	0.34	0.04	-	3.12	0.00	0.01	-
4th morning	8.57	0.80	0.45	0.08	-	4.52	0.00	0.02	-
4th evening	12.17	0.28	0.21	0.02	-	5.81	0.00	0.02	-
Total (µg)	29.81	1.95	1.12	0.16	33.04	15.68	0.00	0.05	15.68
#2									
2nd morning	1.19	0.11	0.07	0.00	-	1.74	0.00	0.01	-
3rd morning	5.00	0.26	0.11	0.00	-	2.18	0.00	0.01	-
4th morning	7.65	0.32	0.21	0.00	-	3.84	0.00	0.01	-
4th evening	9.72	0.18	0.08	0.00	-	4.39	0.00	0.02	-
Total (µg)	23.56	0.87	0.47	0.00	24.90	12.15	0.00	0.04	12.15
#3									
2nd morning	2.00	0.16	0.08	0.00	-	1.48	0.00	0.01	-
3rd morning	4.49	0.27	0.13	0.01	-	3.20	0.00	0.01	-
4th morning	5.41	0.44	0.20	0.01	-	4.22	0.01	0.01	-
4th evening	11.73	0.38	0.17	0.02	-	6.83	0.00	0.03	-
Total (µg)	23.63	1.24	0.58	0.04	25.49	15.73	0.01	0.05	15.74

Much lower concentrations of PTX2 and DTX1 were the dominant toxins in feces collected from each individual scallop (Table 5). These results indicate that toxins not retained in the scallop tissues but are directly excreted to the seawater mostly without metabolic processing. Recoveries of PTXs and DTXs calculated from the sum of excreted and accumulated toxins in scallops against assimilated toxins through *D. fortii* cells were 85%–96% and 75%–87%, respectively (Table 6). Approximately 54%–75% and 52%–70% of assimilated PTX2 and DTX1 were excreted from scallops, respectively. 

**Table 5:** Total amount (×10^−3^) (µg) of toxins in feces.

(ng)	PTX2	PTX1	PTX3	PTX6	PTXs	DTX1	DTX3	OA	DTXs
#1									
2nd morning	114.4	6.2	5.3	7.8	-	17.8	2.0	0.0	-
3rd morning	200.0	9.0	1.1	4.1	-	46.5	6.8	0.0	-
4th morning	124.5	9.7	2.5	5.3	-	28.2	1.3	0.0	-
4th evening	358.8	4.6	0.0	3.3	-	185.2	0.8	0.0	-
Total (ng)	797.7	29.5	8.9	20.2	856.3	277.7	10.9	0.0	288.6
#2									
2nd morning	4.7	0.4	0.0	0.0	-	5.4	2.0	0.0	-
3rd morning	24.0	1.1	0.0	0.0	-	22.7	4.4	0.0	-
4th morning	66.8	3.4	1.2	0.0	-	53.6	1.9	0.0	-
4th evening	152.0	1.2	0.0	0.8	-	73.5	0.3	0.0	-
Total (ng)	247.5	6.1	1.2	0.8	255.6	155.2	8.6	0.0	163.8
#3									
2nd morning	20.5	1.1	0.0	0.0	-	18.9	26.1	0.0	-
3rd morning	17.6	1.1	0.0	0.0	-	11.0	2.3	0.0	-
4th morning	39.7	3.3	0.0	0.0	-	52.7	1.4	0.0	-
4th evening	104.1	2.2	0.0	0.8	-	79.3	0.8	0.0	-
Total (ng)	181.9	7.7	0.0	0.8	190.4	161.9	30.6	0.0	192.5

**Table 6:** Recoveries of PTXs and DTXs calculated from the sum of excreted and accumulated toxins in scallops against provided toxins through *D. fortii* cells.

(µg)	Excreted PTXs	Accumulated PTXs	Total PTXs	Assimilated PTX2	Recovery (%)	Excreted DTXs	Accumulated DTX1	Total DTXs	Assimilated DTX1	Recovery (%)
#1	33.90	9.55	43.45	45.30	95.92	15.97	1.63	17.60	22.93	76.75
#2	25.16	14.33	39.49	46.44	85.03	12.31	5.40	17.71	23.51	75.33
#3	25.69	18.77	44.46	47.54	93.52	15.93	4.97	20.90	24.08	86.79

Recovery (%): (Sum of excreted and accumulated toxins)/assimilated toxins × 100.

## 3. Discussion

Japanese scallops, *Patinopecten yessoensis*, were fed with the toxic dinoflagellate *Dinophysis fortii* to elucidate the relative magnitude of assimilation, accumulation, and metabolism of diarrhetic shellfish toxins (DSTs) and pectenotoxins (PTXs). The cellular content of PTX2 in *D. fortii* was higher than those of DTX1 as reported in natural and cultured *D. fortii* cells [15,26]. In our previous study on cultured *D. fortii*, an increase in the cellular toxin content in exponential growth phase was observed [26]. A similar trend was observed in our present study. It is interesting that *D. fortii* collected in Japan only produce free DTX1 and OA in contrast that *D. acuta* in New Zealand produces several OA diol esters [27]. 

It was reported that the toxicity of scallops contaminated with DSTs determined by mouse bioassay (MBA) was exclusively detected in the digestive gland and the toxicity of gills, mantles, gonads, and adductor muscles were less than detectable levels [1]. Although the higher toxin content of the digestive gland in comparison with other tissues obtained by LC/MS/MS was consistent with the previous study [1], contamination of toxins in other Japanese scallop tissues was confirmed for the first time.

The increase of the DTX1 concentration in the digestive gland samples subjected to alkaline hydrolysis was higher than that obtained by direct quantification of DTX3 (7-*O*-16:0-DTX1) (Figure 4F). This suggests the presence of other DTX3 homologues esterified with other fatty acyl groups such as 18:0 and 20:5 [28]. PTX6 and DTX3 were dominant toxins in the digestive gland of scallops. These results confirm the rapid biotransformation of PTX2 and DTX1 to their respective metabolites and accumulation in the digestive gland of Japanese scallops as reported in our previous studies [17,23]. This is assumed to be a detoxification mechanism within the scallop tissues because the major metabolites (PTX6 and DTX3) have lower toxicities than their parent compounds (PTX2 and DTX1) [1,22,24,29]. 

The higher proportion of PTX2 in the gills in comparison with other tissues is noteworthy. This suggests that PTX2 is selectively retained on the gills and this tissue may take part in some metabolic activities in conversion of PTX2 to other analogues. PTXs may be distributed to other organs for metabolism, while DTX1 appears to be accumulated and metabolized mainly in the digestive gland. PTXs and DTXs are both lipophilic constituents but it is suggested they pass through different metabolic pathways.

Accumulation rates of DTXs in the digestive gland reported in our previous study of scallops fed with natural *D. fortii* was less than 3% [17]. The accumulation of DTXs obtained in our present study was higher than that reported previously (Table 3). The difference in the feeding regimes between the previous and the present studies was the planktonic compositions fed to scallops. In our previous study, *D. fortii* collected from natural seawater was fed to scallops with other phytoplankton although the planktonic composition was dominated by *D. fortii*. In the present study, scallops were fed with uni-algal cultured *D. fortii*. Feeding on other plankton species besides *Dinophysis* may affect accumulation rate of toxins in scallops.

The marked increase in specific toxins in the waters used to house the scallop in the evening of fourth day could be explained by relatively high cell numbers of *D. fortii* fed to the scallops (Table 1 and Table 4). An interesting finding of our present study is that 54%–75% of PTX2 and 52%–70% of DTX1 assimilated by the scallops was directly excreted to the environmental seawater in the form of DTX1 and PTX2 mainly without metabolic processing.

## 4. Experimental Section 

### 4.1. Chemicals

High performance liquid chromatography (HPLC)-grade solvents (acetonitrile, methanol) and analytical-grade solvents (methanol) were purchased from Wako (Osaka, Japan). Analytical-grade reagents (formic acid, ammonium formate) were purchased from Nacalai (Kyoto, Japan). Distilled water was passed through a Milli-Q water purification system (Millipore, Bedford, MA, USA) and used for the preparation of LC mobile phases.

### 4.2. Standard Toxins

Authentic standards of okadaic acid (OA), dinophysistoxin-1 (DTX1), and 7-*O*-palmitoyl (16:0)-dinophysistoxin-1 (DTX3), pectenotoxin-1 (PTX1), pectenotoxin-2 (PTX2), pectenotoxin-3 (PTX3), pectenotoxin-6 (PTX6) were prepared according to previous methods [30]. Toxins were dissolved in HPLC grade methanol to prepare the calibration standards.

### 4.3. Cultures of Dinophysis Fortii

The cryptophyte, *Teleaulax amphioxeia*, and the marine ciliate, *Myrionecta rubra*, were isolated from Inokushi Bay, Oita Prefecture, Japan, in 2007 [31]. The culture of *M*. *rubra* was maintained by weekly re-inoculation into a modified f/2 medium, with the addition of *T. amphioxeia* culture, at 18 °C and under cool-white fluorescent lamps with a 12:12 light:dark (L:D) cycle [32]. The strain of *Dinophysis fortii* was isolated from Hiroshima Bay, Japan (34.16° N, 132.15° E), in 2011. The *M*. *rubra* culture grown until the late logarithmic growth phase (~8.5 × 10^3^ cells/mL) was diluted with fresh culture medium to give an initial concentration of ~2.5 × 10^3^ cells/mL in 150 mL of the culture medium of 250 mL capacity polycarbonate Erlenmeyer flasks (Corning, Corning, NY, USA). Next, 1.5 mL of a *D*. *fortii* culture containing 3.0 × 10^3^ cells (a clonal strain) was added into the *M*. *rubra* culture to give an initial concentration of 20 cells/mL. One hundred µL of the *T*. *amphioxeia* culture (~1.2 × 10^4^ cells) was added into the flasks to give an initial concentration of ~80 cells/mL [32]. The culture of *D*. *fortii* was maintained by re-inoculation into *M*. *rubra* culture at 20 °C. The cell densities of *D*. *fortii* from each culture flask were counted by microscopic observation of living cells. 

### 4.4. Toxin Contamination of Scallops by Feeding with D. Fortii

Four non-toxic scallops (shell length: 8.85 cm ± 0.39) were collected in Funka Bay Hokkaido in January and were acclimated for 24 days in sand filtered seawater at 11–14 °C. The scallops were cleaned and sessile organisms on the shell were removed with a metal brush and scraper. Three individual scallops were separately exposed to cultured *D*. *fortii* for four days in 500 mL static sand filtered seawater in a 1000 mL beaker. Another individual scallop was also kept in a beaker without feeding as a control. Cultured *D*. *fortii* were fed to scallops according to a time table as shown in Table 7. The scallops were fed 15 times through the experimental period. Scallops were sacrificed at 18:30 on the fourth day. Seawater was changed daily and remaining *D*. *fortii* cells in the seawater were counted under a microscope.

**Table 7:** Time table of feeding experiment.

1st day	Three times/14:00, 15:00, 16:00
2nd–3th day	Six times/11:00, 12:00, 13:00, 14:00, 15:00, 16:00
4th day	Six times/11:00, 12:00, 13:00, 14:00, 15:00, 16:00 Sacrificed 18:30

### 4.5. Extraction of DSTs from Scallops

Each dissected tissue (digestive gland, adductor muscle, gill, gonad, mantle, and the remainder: lip, kidney, and foot) was homogenized with nine volumes of methanol-distilled water (9:1, *v*/*v*), and the homogenates were centrifuged at 3000 rpm for 5 min [24]. Supernatants were kept in a freezer until LC/MS/MS analysis.

### 4.6. Extraction of DSTs from D. fortii Cells, Seawater, and Feces

A one mL aliquot of *D. fortii* culture was gently filtered through a glass Pasteur pipette packed with Kimwipes paper and toxins were extracted with 1 mL methanol. Solid phase extraction (SPE) of DSTs and lipophilic toxins from seawaters was carried out according to a previous method [23,33]. Seawater samples were gravity filtered and 40 mL was transferred to a Sep-Pak C18 cartridge column (Waters, Milford, MA, USA) which had been previously washed with 10 mL methanol and water. The cartridge columns were washed with 10 mL water and toxins were eluted with 5 mL methanol. The methanol effluents were evaporated and residues were dissolved in 200 μL of methanol. Toxins in feces collected by gravity filtration of seawater and pipette were extracted with two volumes of methanol after ultrasonic treatment. After centrifugation, the supernatant was evaporated and dissolved in 1 mL of methanol-distilled water (1:1, *v*/*v*).

### 4.7. Hydrolysis of Esterified DTX

Alkaline hydrolysis of digestive gland extracts was carried out according to EU harmonized SOP LIPO LCMSMS Ver. 4. For the hydrolysis of esterified toxins in the scallop extracts or *D. fortii* extracts, 125 μL of 2.5 M NaOH solution was added to a 1 mL aliquot of a methanolic extract of each tissue. The mixture was kept at 80 °C for 30 min and neutralized with 125 μL of 2.5 M HCl. The hydrolysis samples were directly analyzed by LC/MS/MS.

### 4.8. LC/MS/MS Analysis of DSTs and PTXs

OA, DTX1, 7-*O*-16:0-DTX1 (DTX3), PTX1, PTX2, PTX3, PTX6 in sample extracts were analyzed and quantified by liquid chromatography/tandem mass spectrometry (LC/MS/MS) as reported in our previous methods [15,24]. Single analysis was carried out for each sample extracts. A model 1100 liquid chromatograph (Agilent, Palo Alto, CA, USA) was coupled to a hybrid triple quadrupole/linear ion trap mass spectrometer Q Trap™ (PE-SCIEX, Thornhill, ON, Canada). Separations were performed on Quicksilver cartridge columns (50 mm × 2.1 mm i.d) packed with 3 µm Hypersil-BDS-C8 (Keystone Scientific, Bellefonte, PA, USA) maintained at 20 °C. Eluent A was water and B was acetonitrile–water (95:5), both containing 2 mM ammonium formate and 50 mM formic acid. Linear gradient elution from 20% to 100% B was performed over 10 min and then held at 100% B for 15 min, followed by re-equilibration with 20% B (13 min). The flow rate was 0.2 mL/min and the injection volume was 10 µL. The LC effluent was introduced into a TurboIonSpray interface without splitting. High-purity air heated to 500 °C was used as the nebulizing gas. Multiple reaction monitoring (MRM) LC/MS/MS analysis for toxins was carried out using [M-H]^−^ as the target parent ions in Q1 and particular fragment ions of each toxin in Q3, with a dwell time of 100 msec for each analogue as follows. OA: *m/z* 803.5 > 255.3; DTX1: *m/z* 817.5 > 255.3; 7-*O*-16:0-DTX1 (DTX3): *m/z* 1055.7 > 255.3; PTX1: *m/z* 873.5 > 137.2; PTX2; *m/z* 857.5 > 137.2; PTX3: *m/z* 871.5 > 137.2; PTX6: *m/z* 887.5 > 519.4.

## 5. Conclusions

Detailed DSTs and PTXs accumulation kinetics in Japanese scallops fed with the toxic dinoflagellate *D. fortii* was clarified in our present study. Toxins were mainly accumulated in the digestive gland although low levels of toxins were detected in the gills, mantles, gonads, and adductor muscles. The toxin concentrations in tissues were in the order of: digestive gland > gill > mantle > gonad > adductor muscle. Because the amount of toxin in the adductor muscle was extremely low, the validity of the practise of evisceration of Japanese scallops contaminated with DSTs (implemented since 1980 in Japan) was confirmed. The accumulation efficiencies of PTXs and DTXs in the scallops, (accumulated toxins *versus* assimilated toxins) were 21%–39% and 7%–23%, respectively. The higher the accumulation efficiency of PTXs in comparison to OA/DTXs could partly contribute to the higher concentrations of PTXs in toxin profiles of Japanese scallops. An interesting finding of our present study is that 54%–75% of PTX2 and 52%–70% of DTX1 assimilated by the scallops was directly excreted to the environmental seawater in the form of DTX1 and PTX2 mainly without metabolic processing. 

There is a concern about the limited numbers of scallops (*n* = 3) used. While the patterns do seem quite consistent between these, using a larger sample would provide a stronger basis for analysis and conclusions.
